# Recruiting Medical Students for a First Responder Project in the Social Age: Direct Contact Still Outperforms Social Media

**DOI:** 10.1155/2020/9438560

**Published:** 2020-06-01

**Authors:** David Marx, Robert Greif, Mike Egloff, Yves Balmer, Sabine Nabecker

**Affiliations:** ^1^Department of Anaesthesiology and Pain Medicine, Bern University Hospital and University of Bern, Bern, Switzerland; ^2^ERC ResearchNET, Bern, Switzerland; ^3^School of Medicine, Sigmund Freud University Vienna, Vienna, Austria

## Abstract

**Introduction:**

Efficient recruitment of first responders (FRs) is crucial for long-term success of any FR project. FRs are laypersons who are trained in cardiopulmonary resuscitation (CPR), medical professionals, and firemen, police officers, and other professions with a duty of help. As social media are widely used for rapid communication, we carried out a prospective observational study to test the hypothesis that recruitment of FRs via social media is more efficient than recruitment via direct face-to-face contact.

**Methods:**

Following ethics committee agreement, we informed 600 medical students about becoming FRs when they attended a didactic lecture about the FR project or during their mandatory CPR-course. Furthermore, recruitment was opened to medical students through Facebook, which accessed ∼1,000 medical students to see if they expressed interest in becoming FRs. All of the recruited students successfully completed the FR training. We then used an online questionnaire to ask these students how they had been recruited.

**Results:**

Out of 63 registered student FRs, 59 responded to the online questionnaire. Overall, 15.3% of these FR students were recruited via social media. The majority (78.0%) were recruited through direct contact.

**Conclusions:**

Despite widespread use of social media, over three-quarters of these medical students were recruited to the FR project via direct personal contact. This suggests that the advantage of a larger reachable population using social media does not outweigh the impact of personal contact with experts.

## 1. Introduction

The term “first responder (FR)” is used for people who are dispatched to a cardiac arrest who can perform cardiopulmonary resuscitation (CPR). There are different standards for such FRs. In some countries, laypersons trained in CPR can become FRs [1–3]. In other countries, either a medical background is required (e.g., Emergency Medical Services personnel, nurses, and doctors), or professionals with the duty of help are FRs (e.g., firemen, police officers, and security personnel) [4–7]. In some countries, FR systems combine these groups [8–11].

Early CPR and defibrillation have been shown to be effective to improve outcome after out-of-hospital cardiac arrests [6–13]. However, early CPR by trained CPR providers is critical, as the 1-month survival for patients suffering out-of-hospital cardiac arrests is higher if the rescuers are trained in CPR, compared to untrained bystanders [14]. Early defibrillation is associated with increased survival rates and increased rates of survival with good neurological outcomes [3, 8]. However, a recent Cochrane review on the dispatch of community FRs concluded that despite increased rates of early CPR and defibrillation, it is unclear whether or not FR dispatch increases the overall survival of out-of-hospital cardiac arrests [15]. Despite clear evidence, FR systems are being increasingly implemented, and recruitment of FRs is crucial to keep the FR system alive.

It is said that we are now living in the “social age,” where technology-enhanced collaboration via social media is the preferred way of communication amongst the youth of today [16]. In 2016, nearly 98% of people aged 18–24 years in the USA used social media regularly [17]. Amongst healthcare providers, 88% use social media regularly [18]. In 2018, Leary et al. showed that the use of a small number of Twitter bloggers can significantly enhance the social impact of a single event [19]. In contrast, a comparison in the recruitment of health research participants between Facebook and traditional recruitment methods did not find any differences in the response rates [20]. However, a systematic review has suggested that using Facebook to recruit research participants improved the selection of participants and reached participants in more remote regions [21].

Currently, there is no evidence available on the most effective recruitment strategies for laypersons to become FRs. Therefore, this prospective observational study evaluated the efficiency of social media compared to direct face-to-face contact to recruit undergraduate medical students into the cantonal FR project in Bern, Switzerland. The primary hypothesis of this study was that recruitment via social media is more effective than recruitment via direct contact.

## 2. Materials and Methods

The Cantonal Ethics Committee of Bern, Switzerland (Basec no. 2019-00666), agreed on the anonymous online survey and reporting of these data.

In February 2017, medical students at the University of Bern attended didactic lectures about the local FR project. During the annual Basic Life Support refresher courses (4th year), the mandatory Immediate Life Support courses (5th year), and the facultative Advanced Life Support courses (6th year), the instructors of the Bern Simulation- and CPR-Centre informed all medical students about the FR project. In all, 600 medical students were reached via this direct face-to-face contact.

Also in February 2017, a Facebook message was posted on the official medical student group of the University of Bern, to also introduce the FR project, which included the slides of the PowerPoint presentation from the lecture on the FR system (see above). Approximately 1,000 medical students were reached through this Facebook group. The total number of students in the medical school at the University of Bern is 2,100.

During the study period from February to April 2017, all of the University of Bern medical students were invited to become FRs through participation in the local FR training. This included the following:

1. A 45 min introduction of the App-based alarm system, the response duties, and the reporting and feedback required of FRs, given by the local Emergency Medical Service in collaboration with clinical experts from the Bern Simulation- and CPR-Centre
2. A 1 h small-group Basic Life Support refresher course that included the use of automated external defibrillators, to ensure high-quality CPR skills

Following their successful completion of this FR training, the medical students were eligible to register as FRs and were asked to fill in an online questionnaire (Doodle AG, Zurich, Switzerland) that asked how they were recruited to participate in the FR training. The given options were as follows: (1) social media (Facebook post or other social media), (2) direct contact (didactic lecture or face-to-face communication by CPR instructors), and (3) other sources. Multiple answers were allowed in the survey.

## 3. Results

During the study period from February to April 2017, between 1,000 and 1,600 medical students were reached via social media and direct face-to-face contact (there might have been overlap of students reached by both methods). In all, 77 (between 4.8 and 7.7%) of the medical students who were reached signed up for the FR training, although two were excluded because they were in the 2nd year of their medicine studies.

Of the 75 medical students who participated in the FR training, 11 (14.7%) did not complete it and 1 (1.3%) refused to become a FR. In total, 63 (84.0%) became registered FRs: 43 (68.3%) were female, and the mean age was 24 ± 1 years. Fifty-nine (93.7%) of them answered the online questionnaire about their route to recruitment (Table 1).

The defined hypothesis was not met, as only 15.3% of the FR students were recruited via social media. The majority (78%) were recruited through direct contact (Table 1). Only one student indicated that he/she was recruited via social media and direct contact.

## 4. Discussion

This survey reports on the recruitment route of undergraduate medical students to become local FRs in the Canton of Bern, Switzerland. Although we reached 1,000 to 1,600 medical students by social media and direct contact, only 3.9 to 6.3% finally became FRs. Of those who responded to the online questionnaire after their FR training, 78% were recruited via direct face-to-face contact and 15.3% via social media.

One main concern for FR systems is how to effectively recruit FRs to keep the system alive. It has been well documented that social media are widely used not only among young people but also among healthcare professionals [17, 18]. Therefore, it was a reasonable assumption that recruitment of FRs via social media would probably be more effective than recruitment via direct face-to-face contact. However, the reality in the present survey was contrary to this hypothesis. This survey suggests that direct face-to-face contact is still an effective way to recruit medical students into a local FR project.

A previous study reported that recruitment of participants to health research projects via social media was comparable to the more traditional ways of recruitment [20]. One reason for the high recruitment rate via direct contact in the present survey was probably that the two experienced Bern Simulation- and CPR-Centre Basic Life Support instructors taught the didactic lecture as “peer teachers.” Thus, they were both medical students and were well known to most of the medical students who attended their lecture. This might have had an influence on the rate of recruitment.

In comparison, a systematic review on recruitment of health research participants suggested that social media use might improve participant selection and reach more participants in remote areas better than traditional recruitment methods [21]. The main difference in recruiting participants for a FR project is that FRs can help others over a long period. It was not necessary to select special populations here, but to recruit as many people as possible. The enhanced ability to recruit participants in remote areas might be beneficial though. All FRs recruited in this project are situated in the Canton of Bern, Switzerland, which is an area of about 6,000 km^2^. However, we do not know if the FRs act in urban or remote areas. Therefore, we cannot comment on the particular benefits of the selection of participants in remote areas.

Recruitment via social media has its limitations, though, because only participants with Internet access can participate. To become a Bern FR, there is the need to have a smartphone with Internet access, as the alarms to dispatch the FRs are App-based. Therefore, the theoretical limitation of reaching participants only by Internet was not part of the argument here.

In this survey, 71% of the recruited medical students were female. This indeed reflects the current proportion of female medical students at the University of Bern, which is >70%.

It might be suggested that medical students will show a higher response rate for participation in FR-training than laypersons due to their moral or professional responsibility to act as rescuers. However, in the present survey, only 3.9 to 6.3% of the medical students reached actually became FRs. The reason for this might be linked to the low social media responses, where one Facebook post was used to recruit the medical student to the FR training. Leary et al. demonstrated that smaller numbers of Twitter bloggers were able to significantly raise the social impact of one single event [19]. Therefore, more Facebook posts might have generated different results.

Overall, the present survey suggests that FR projects should acknowledge the importance of direct face-to-face contact for recruiting new FRs.

## 5. Conclusions

The assumed advantage of reaching a larger population via social media has been outweighed by the direct face-to-face contact here. This is illustrated by the 78% of the medical students in our survey who were recruited via direct face-to-face contact, despite their daily use of social media.

## Figures and Tables

**Table 1:** Characteristics and recruitment route of the participants in the first responder training who responded to the online questionnaire.

Characteristics	Participants (*n*)	59
	Female, *n* (%)	42 (71.2)
	Age, years (mean ± standard deviation)	24 ± 1

Recruitment route	Recruitment via social media, *n* (%)	9 (15.3)
	Recruitment via direct contact, *n* (%)	46 (78.0)
	Recruitment via social media and direct contact, *n* (%)	1 (1.7)
	Recruitment via other sources, *n* (%)	3 (5.1)

## Data Availability

The datasets used and/or analysed during the current study are available from the corresponding author on reasonable request including a research question.
